# Ulcerative Colitis Gone Rogue: A Case of Complement-Mediated Thrombotic Microangiopathy in Inflammatory Bowel Disease

**DOI:** 10.7759/cureus.78447

**Published:** 2025-02-03

**Authors:** Eugene K Yeboah, Aye M Thida, Ramtin Moradi, Dedipya Bhamidipati, Prashil Dave, Muhammad Azhar, Mary Mallappallil, Isha Puri

**Affiliations:** 1 Internal Medicine, State University of New York Downstate Health Sciences University, Brooklyn, USA; 2 Hematology and Oncology, State University of New York Downstate Health Sciences University, Brooklyn, USA; 3 Nephrology, State University of New York Downstate Health Sciences University, Brooklyn, USA; 4 Nephrology, New York City Health and Hospitals (NYCHHC) Kings County Hospital Center, Brooklyn, USA

**Keywords:** adamts-13 deficiency, alternate pathway complement, atypical hus, complement dysregulation, complement-mediated thrombotic microangiopathy, eculizumab, thrombotic microangiopathy (tma), thrombotic thrombocytopenic purpura (ttp)-like syndrome

## Abstract

We present an unusual case of complement-mediated thrombotic microangiopathy (formerly known as atypical hemolytic uremic syndrome) associated with inflammatory disease in a young patient. A 26-year-old male patient with no significant past medical history presented to our emergency department with a four-week history of diffuse, moderate, cramping, non-radiating abdominal pain with no known aggravating or relieving factors. Abdominal pain was associated with nausea, vomiting, and bloody stools. His physical examination revealed pale conjunctiva, tachycardia, and mild tenderness in the lower abdomen. The patient's laboratory results indicated severe anemia with a hemoglobin level of 2.9 g/dL, an elevated white blood cell count of 52.86 K/uL, a low platelet count of 107 K/uL, and evidence of acute kidney injury, with a blood urea nitrogen level of 87.0 mg/dL and a serum creatinine level of 8.32 mg/dL. Further work-up showed hemolysis, characterized by low haptoglobin levels, elevated lactate dehydrogenase, and a positive direct Coombs test for both anti-IgG and anti-C3 antibodies. A computed tomography angiogram (CTA) of the abdomen and pelvis showed pancolitis. Severe inflammation was noted during a flexible sigmoidoscopy, and pathology results revealed chronic inflammation/chronic colitis. A renal biopsy performed showed thrombotic microangiopathic changes with complement deposition. The patient was started on eculizumab, which ultimately resulted in improvements in anemia, thrombocytopenia, and renal function. Our case stands out as the complexity of the diagnosis warrants awareness of complement-mediated thrombotic microangiopathy (TMA). The introduction of eculizumab, a terminal complement blockade therapy, has revolutionized the management of complement-mediated TMA, as early initiation of eculizumab treatment has shown significant reductions in disease progression to end-stage kidney disease and its related complications.

## Introduction

Thrombotic microangiopathy (TMA) is a term used to describe histologic lesions in the endothelium found in several medical conditions [1]. The first description of TMA is attributed to Dr. Eli Moschowitz in 1924 [2]. In 1952, Professor William St Clair Symmers suggested TMA was characterized by pathognomonic disseminated lesions that occurred only in microscopic vessels [1,3]. A few years later, hemolytic uremic syndrome (HUS) was described. The pathogenesis of TMA was, however recently established as endothelial cell injury associated with alterations in factors that affect angiogenesis, coagulation, platelet activation, and complement function [1,3]. TMA syndromes are described by specific clinical characteristics, including thrombocytopenia, microangiopathic hemolytic anemia (MAHA), and pathologic evidence of endothelial cell damage, and all these manifesting as ischemic end-organ injuries [1,3].

Numerous clinical scenarios, including but not limited to infection, pregnancy, malignancy, autoimmune disease, and medications, have been recognized as triggering factors for TMA initiation [1]. The overlapping clinical presentations can hamper differential diagnosis of the underlying pathogenesis despite recent advances in understanding the mechanisms of several types of TMA syndrome [1,3]. In this case report, we present a previously healthy 26-year-old male diagnosed with complement-mediated TMA (formerly known as atypical hemolytic uremic syndrome) who manifested significant renal impairment potentially linked to inflammatory bowel disease (IBD).

## Case presentation

A 26-year-old male presented with diffuse, moderate, vague, non-radiating abdominal pain with no known aggravating or relieving factors. Abdominal pain was associated with nausea, vomiting, and bloody stools but not hematemesis. The patient denied any fever, chills, cough, sputum production, or dysuria. The patient had reduced urine volume and frequency two weeks prior to reporting to us but did not notice any change in urine color. The patient was seen in a different facility by a gastroenterologist about a month prior to reporting to our facility and was planned for outpatient lower endoscopy to evaluate for possible inflammatory bowel disease. The patient's home medications were famotidine and fiber supplements. The patient did not have a personal or family history of colorectal cancer. The patient did not have a history of smoking but had a history of drinking alcohol with four standard units per week and a history of lead exposure as a child.

On physical examination, the patient was weak and acutely ill-looking but was awake, alert, and oriented to place and time. He was afebrile and tachycardic with a heart rate of 119 bpm and a respiratory rate of 16 cpm, SpO2 100% on room air, blood pressure 114/62 mmHg, BMI 17.18 kg/m2, weight 51.3 kg and height 1.7270 m. The patient was anicteric but with pale conjunctiva with dry mucous membranes. A chest was clinically clear, and the abdomen was soft, moved with respiration, non-tender, no guarding, and no palpable masses. A digital rectal examination showed brown stool on the examining finger with no palpable masses and no blood seen on the examining finger. The patient's laboratory testing is summarized in Table 1.

**Table 1:** Summary of the patient's lab work DNA - deoxyribonucleic acid; PCR - polymerase chain reaction

Parameter	Patient values	Reference range
Comprehensive metabolic panel		
Sodium	133mmol/L	136-145 mmol/L
Potassium	2.7mmol/L	3.5-5.1 mmol/L
Calcium	7.0mg/dL	8.2-10.0 mg/dL
Chloride	96mmol/L	98-107 mmol/L
Magnesium	1.9mg/dL	1.9-2.7 mg/dL
Creatinine	8.32mg/dL	0.7-1.3 mg/dL
Blood urea nitrogen	87.0mg/dL	7-25 mg/dL
Carbon dioxide	22mmol/L	21-31 mmol/L
Glucose	94mg/dL	70-99 mg/dL
Anion gap	15mmol/L	10-20 mmol/L
Estimated glomerular filtration rate	8.4ml/min/1.73m²	>60 ml/min/1.73m²
Liver function test		
Total bilirubin	0.7mg/dL	0.3-1.0 mg/dL
Albumin	1.9g/dL	3.5-5.7 g/dL
Total protein	5.7g/dL	6.0-8.3 g/dL
Aspartate aminotransferase	65U/L	13-39 U/L
Alanine aminotransferase	69U/L	7-52 U/L
Alkaline phosphatase	122U/L	34-104 U/L
Complete blood count		
Hemoglobin	2.9g/dL	14.0-18.0 g/dL
White blood count	52.66k/μL	3.5-10.8 k/μL
Platelet	107k/μL	130-400k/μL
Hematocrit	8.4%	42.0-52.0%
Schistocytes	Numerous	Negative
Urinalysis		
pH	5.0	5.0-8.0
Specific gravity	1.014	1.005-1.030
Urine glucose	Negative	Negative
Urine blood	Moderate	Negative
Urine creatinine	80.64 mg/dL	20-320 mg/dL
Urine protein	100mg/dL	Negative
Urine nitrite	Negative	Negative
Leucocyte esterase	Negative	Negative
White blood cells (Urine)	10.6 /hpf	0-5/hpf
Urine cast	3-5/Ipf	0-2/Ipf
Coagulation		
Prothrombin time	11.7sec	10.8-13.7 sec
Activated partial thromboplastin time	26.0sec	25-35 sec
International normalized ration	1.0	<1
Haptoglobin	<10mg/dl	30-300mg/dl
Lactate dehydrogenase	1460U/L	135-225U/L
Fibrinogen	380mg/dl	200-393mg/dl
Glucose-6-phosphate dehydrogenase	20.2U/g Hgb	7.0-20.5U/g Hgb
Von Willibrand factor activity	261%	45-133%
Anemia workup		
Iron (Fe)	44 μg/dL	50-212 μg/dL
Total iron binding capacity	86 μg/dL	240-450 μg/dL
Ferritin	1048ng/mL	16.0-294.0 ng/mL
Percent saturation	51%	20-55%
Glomerulopathy work up		
Cryoglobulin	Negative	Negative
Complement (C3) levels	40mg/dl	86-184mg/dl
Complement (C4) levels	25mg/dl	20-58mg/dl
Complement total (CH50)	17	42-95U/ml
Alternative complement (AH50)	<10	>46
Infectious workup		
Covid 19 PCR	Negative	Negative
Influenza virus	Negative	Negative
Human immunodeficiency virus 1/2 antigen/antibodies	Negative	Negative
Hepatitis A	Non-reactive	Non-reactive
Hepatitis B surface antigen	Non-reactive	Non-reactive
Blood culture	No growth	No growth
Gastrointestinal stool polymerase chain reaction panel	Negative	Negative
Stool *Clostridium difficile* toxin	Negative	Negative
Shiga-like *Escherichia coli*	Negative	Negative
Fecal calprotectin	Positive	Negative
Shigella	Negative	Negative
Tuberculosis QuantiFERON Gold	Negative	Negative
Autoimmune workup		
Antinuclear antibody (ANA)	Negative	Negative
Anti-double stranded DNA	<12 IU/ml	<29 IU/ml
Anti-smith antibodies	Negative	Negative
Rheumatoid factor	Negative	
Scleroderma antibodies (SCL 70)	Negative	Negative
Anti-ribonuclear protein	Negative	Negative
Perinuclear anti-neutrophil cytoplasmic antibodies (p-ANCA)	Negative	Negative
Cytoplasmic antineutrophil cytoplasmic antibodies (c-ANCA)	Negative	Negative
Antineutrophil cytoplasmic antibodies (ANCA)	Indeterminate	Negative

Imaging

**Figure 1:**
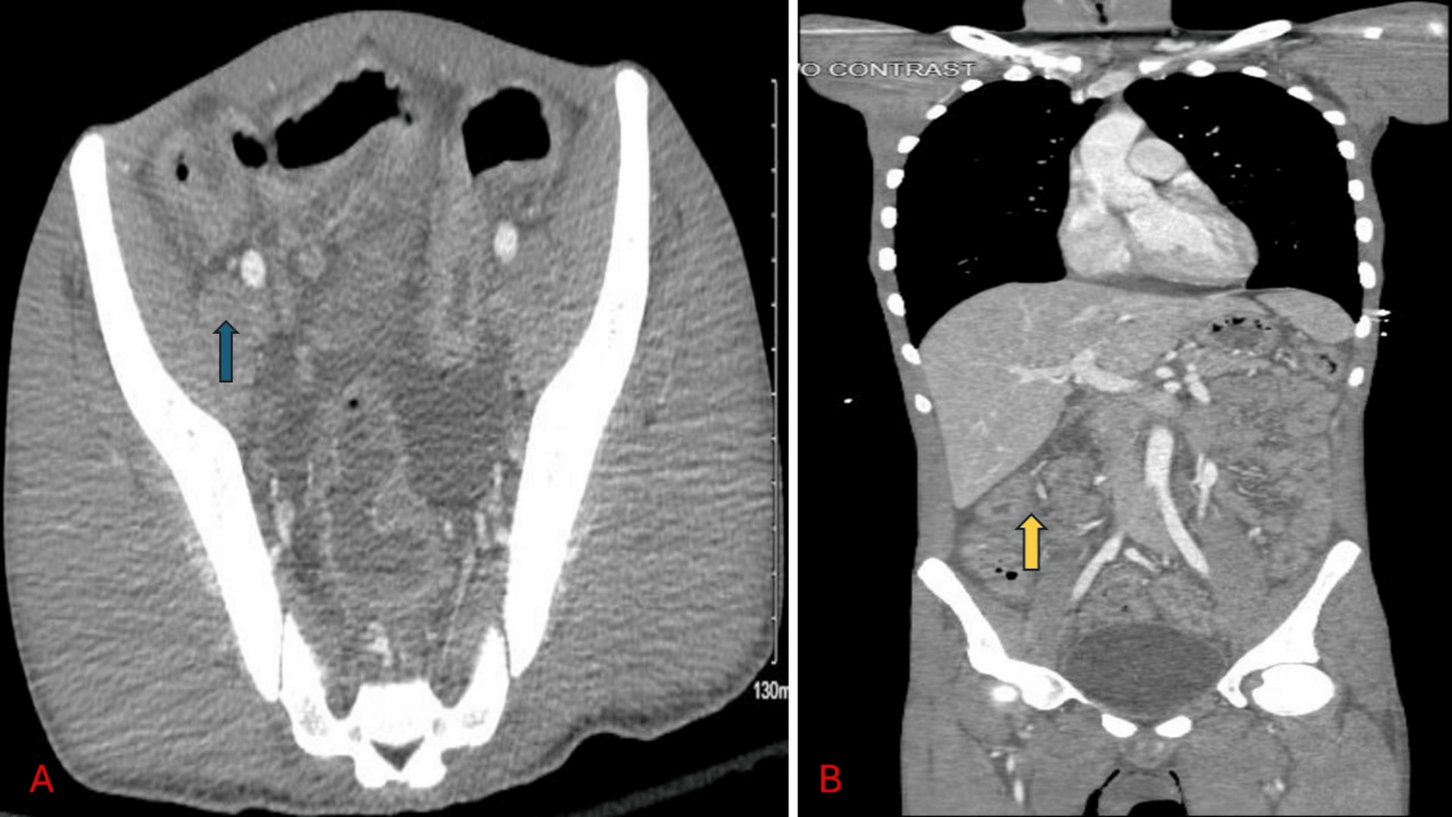
Computed tomography (CT) scan of abdomen and pelvis of the patient A) Blue arrow in A shows colon wall enhancement with haziness and thickening which is consistent with possible inflammation, most likely colitis; B) Yellow arrow in B shows ascending colon wall enhancement and thickening suggestive of colitis

Figure 1 is a computed tomography (CT) scan of the abdomen and pelvis of the patient. It showed colon wall enhancement with haziness and thickening which is consistent with possible inflammation.

Sigmoidoscopy

**Figure 2:**
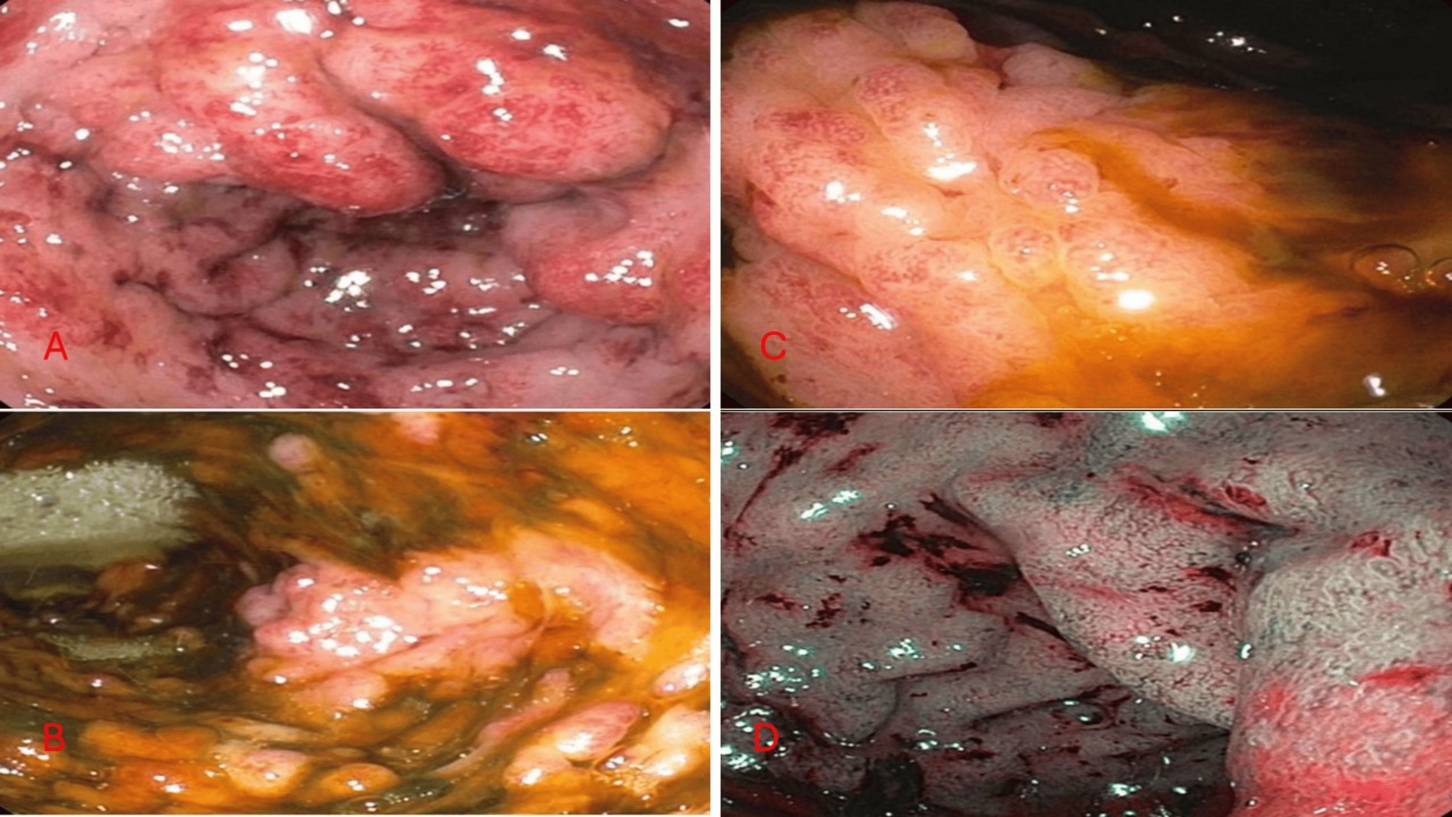
Sigmoidoscopy of the patient A) Sigmoid colon showing diffuse severe inflammation characterized by congestion (edema), erythema, and loss of vascularity found in sigmoid colon. No exudates, no friability, and no pseudomembranous. B) Transverse colon showing diffuse severe inflammation characterized by congestion (edema), erythema, and loss of vascularity. C) Left colon showing diffuse severe inflammation characterized by congestion (edema), erythema and loss of vascularity. The biopsy showed colonic mucosa with mild chronic active inflammation, focal crypitis, and glandular distortion. Lymphoid aggregates were also observed. D) Rectum showing diffuse severe inflammation characterized by congestion (edema), erythema, and loss of vascularity were found in the rectum. The biopsy showed colonic mucosa with lymphoid aggregates.

Figure 2 is a sigmoidoscopy of the patient. It showed diffuse inflammation characterized by edema, erythema, and loss of vascularity. Immunostains for CD3, CD21, and PAX5 were performed and were consistent with a reactive process.

Kidney biopsy

**Figure 3:**
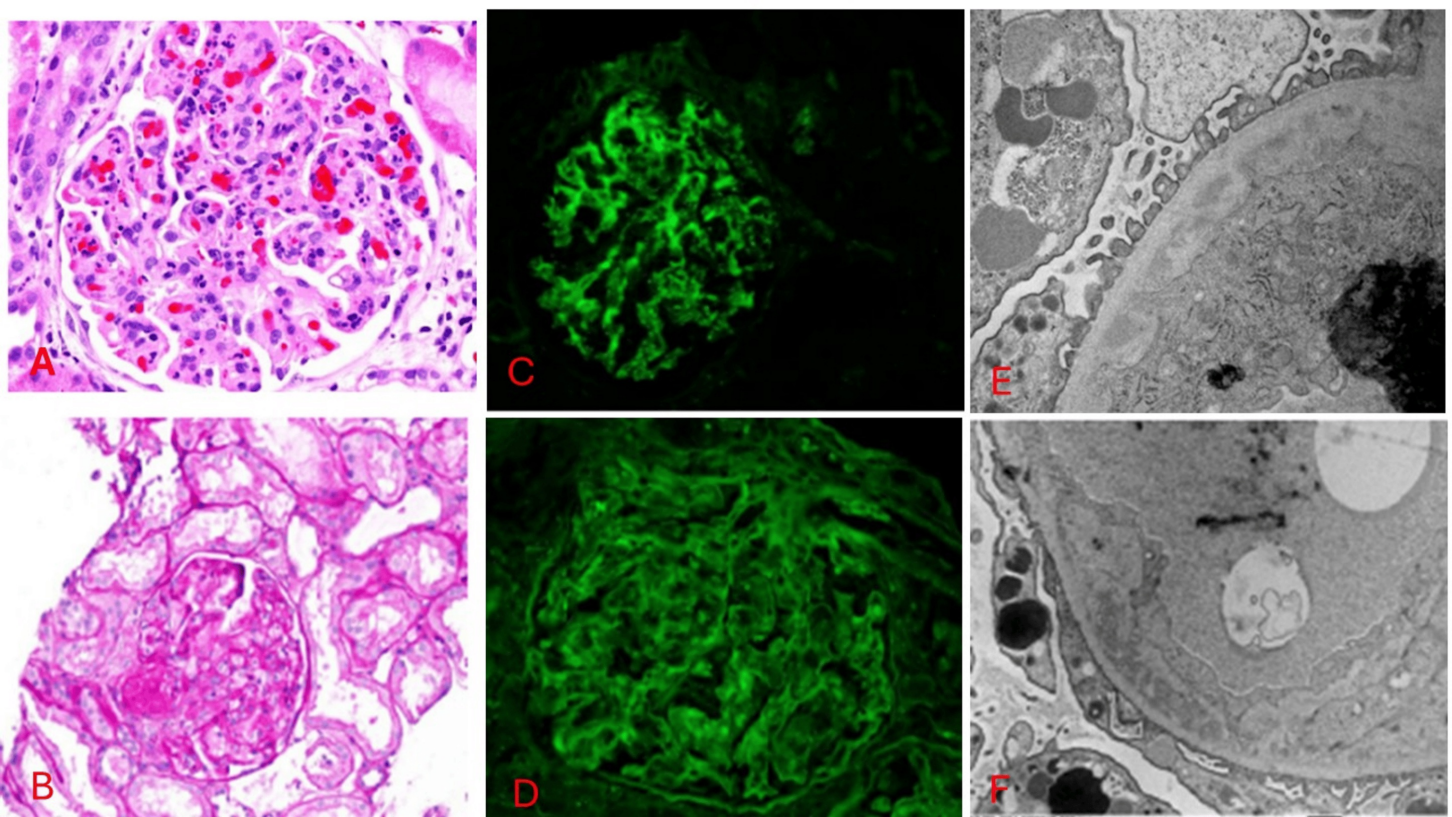
Kidney biopsy findings on light microscopy, immunofluorescence and electron microscopy A) Light microscopy showing glomeruli with diffuse tuft inflammation, frequent polymorphonuclear cells, few mononuclear interstitial inflammatory cells, tuft karyorrhectic debris, foci of tuft schistocytes, and hilar thrombi. There are no glomerular crescents, interstitial fibrosis, tubular atrophy, arteriosclerosis, or arteriolosclerosis in the arteries. There are few pre-glomerular arteriolar thrombi. B) Light microscopy showing Hilar thrombus and tuft inflammation. C) Direct Immunofluorescence showing glomeruli with granular capillary wall and mesangial staining with antiserum specific for C3(3+). The glomeruli have no staining with antisera specific for IgA, IgG, IgM, C1q, kappa light chains, or lambda light chains. There are no significant extraglomerular staining. There is weak tubulointerstitial fibrinogen reactivity. It also shows diffuse segmental podocyte cytoplasmic protein reabsorption droplet reactivity with antiserum specific for albumin, IgG, IgA, kappa light chains, and lambda light chains. D) Direct Immunofluorescence H&E-stained showing negative IgG E) Electron microscopy showing tuft of glomeruli segmental inflammatory cells, small mesangial matrix, and segmental subendothelial immune complex electron-dense deposits. There are segmental visceral epithelial foot process effacement, endothelial fenestrations, and podocyte cytoplasmic vacuoles. There are no endothelial cell luminal cytoplasmic extensions or endothelial tubulovesicular inclusions. It shows thin-walled peritubular capillaries F) Electron microscopy showing capillary wall deposits.

Figure 3 shows a kidney biopsy with immunofluorescence of our patient. Biopsy confirmed thrombotic microangiopathic changes as well as complement deposition.

The patient was managed by a multidisciplinary team involving nephrologists, hematologists, infectious disease specialists, and gastroenterologists. During the hospitalization, the patient's thrombocytopenia, and anemia progressively worsened, necessitating transfusion support, and there was continued deterioration in renal function. Testing for Shiga toxin returned negative, and ADAMTS-13 activity was measured at 43.1%, suggesting a diagnosis of complement-mediated hemolytic uremic syndrome (HUS). He was first started on ceftriaxone and metronidazole for possible infectious colitis before making a diagnosis. The patient was initiated on steroid therapy a few days prior to renal biopsy, which resulted in a reduction in transfusion frequency. Renal biopsy findings corroborated the diagnosis of acute complement-mediated TMA. The patient received an eculizumab induction dose of 900 mg weekly for one month, followed by 1.2 g at week five and, thereafter, 1.2 g every two weeks. Prior to starting eculizumab, the patient was immunized against meningococcus and started on prophylaxis with penicillin. Complete genetic panel testing for ADAMTS13 gene mutations, complement component 3 (C3), membrane cofactor protein (CD46), decay accelerating factor (CD55), membrane inhibitor of reactive lysis (CD59), complement factor B (CFB), complement factor H (CFH), complement factor I (CFI), diacylglycerol kinase epsilon (DGKE), inverted formin-2 (INF2), metabolism of cobalamin associated C (MMACHC), plasminogen (PLG) and thrombomodulin (THBD) showed no pathologic variants.

**Figure 4:**
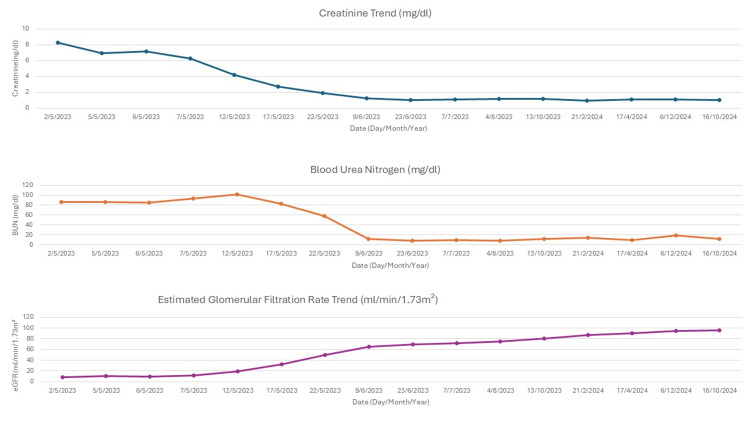
Trend of kidney function Creatinine reference range: 0.7-1.2mg/dl; Blood urea nitrogen (BUN) reference range: 6.0-20.0mg/dl; Estimated glomerular filtration rate (eGFR) reference range: >60ml/min/1.73m²

Figure 4 shows the progressive recovery of kidney function after initiating treatment. The bloody bowel stool resolved, and the anemia resolved. Eculizumab was eventually switched to long-acting ravulizumab. The patient tolerated the medication with no reported side effects. The patient's IBD improved with no flares, and kidney function improved despite not starting dialysis. The patient remained in clinical remission with no active colitis on repeat endoscopic examination. The patient continued to be under regular follow-up by a multidisciplinary team involved in his care.

## Discussion

Thrombotic microangiopathy (TMA) is a term used to describe the histologic lesions in the endothelium found in numerous medical conditions [1]. TMA is characterized by distinct clinical, laboratory, and pathological features [1]. The laboratory features of TMA include microangiopathic hemolytic anemia (MAHA), which is characterized by fragmented erythrocytes (e.g., schistocytes or helmet cells), thrombocytopenia, elevated levels of lactate dehydrogenase and abnormal laboratory results related to TMA-mediated organ damage (e.g., increased creatinine level and hematuria or proteinuria) [1-4]. Diverse disease entities can all lead to TMA, but the key disorders of thrombotic microangiopathy (TMA) include atypical hemolytic uremic syndrome (also known as complement-mediated thrombotic microangiopathy), thrombotic thrombocytopenic purpura, and Shiga toxin-associated HUS [5]. The overlapping in symptomatology can make it difficult to distinguish among the various disorders that can cause TMA [5]. Our patient presented with a month's history of gastrointestinal symptoms preceding the acute kidney injury and hemolytic anemia, and the workup found the patient to have complement-mediated thrombotic microangiopathy associated with inflammatory bowel disease. 

In addition to the classical clinical triad of MAHA, thrombocytopenia and acute kidney injury, extrarenal complications involving the central nervous (drowsiness, seizures, encephalopathy, cortical blindness), cardiovascular (cardiomyopathy, myocardial infarction, heart failure), pulmonary (pulmonary hemorrhage), gastrointestinal (pancreatitis, intestinal bleeding), and skeletal system (rhabdomyolysis) are common and can occur in up to 20% of TMA cases [4]. An atypical classification of the hemolytic uremic syndrome is usually defined with origins unrelated to cobalamin deficiency, streptococci, Shiga toxin-producing bacteria, or other infections such as influenza A, H1N1, and HIV [4-11]. 

Currently, distinguishing between complement-mediated thrombotic microangiopathy and secondary TMA syndromes remains a daunting task [1]. The two conditions have similar manifestations but different treatment strategies [1]. Whereas most secondary TMA syndromes can be managed by the removal of triggering factors, atypical hemolytic uremic syndrome requires correction of both the complement dysregulation with a specific treatment as well as the removal of provoking factors [1]. Timely diagnosis of complement-mediated thrombotic microangiopathy is imperative due to different pathophysiology and therapy. Therapy must be initiated early to prevent end-organ damage [5]. Difficulties in differential diagnosis of thrombotic microangiopathies do still exist but soluble C5b-9 has already been introduced in diagnostic algorithms of complement-mediated thrombotic microangiopathies representing a widely accessible marker [12]. In addition, other cellular-based assays have been described, such as the modified Ham test and an ex vivo assay measuring complement attack on endothelial cells [12].

Eculizumab is a recombinant, humanized, monoclonal immunoglobulin that acts on complement component 5 (C5) and prevents the cleavage of C5 to C5a and C5b [1,13]. Both eculizumab and plasmapheresis are approved treatment modalities for atypical HUS but not secondary TMA syndromes [13-15]. We observed an improvement in kidney function after the initiation of steroids and eculizumab while hemolysis was still active.

To our knowledge, few cases of complement-mediated thrombotic microangiopathy associated with inflammatory bowel disease have been reported [16-19]. Our case will add to the few reported cases and existing knowledge of complement-mediated thrombotic microangiopathy associated with IBD. As our understanding of complement-mediated TMA advances, we will be able to rapidly identify and treat such a devastating disease process. With this case, we wanted to emphasize that CM-TMA is a heterogeneous spectrum of disease that can be acquired or congenital, contrasting with the previous notion of limited well-defined syndrome.

## Conclusions

This case emphasizes the need to consider complement-mediated TMA in patients with IBD presenting with hematologic abnormalities and renal impairment. Eculizumab remains a primary treatment for complement-mediated TMA; however, effective management may require individualized dosing and close monitoring during active IBD. This case contributes to the recognition of IBD as a potential secondary cause of complement-mediated TMA, underscoring the importance of clinician awareness to optimize patient outcomes. Further research is essential to clarify the mechanisms linking IBD and complement-mediated TMA and to develop guidelines for long-term management, particularly regarding dosing strategies during disease flares.
